# RETRACTED: Atta et al. Synthesis of Silver Nanoparticles by Green Method Stabilized to Synthetic Human Stomach Fluid. *Molecules* 2014, *19*, 6737–6753

**DOI:** 10.3390/molecules31203677

**Published:** 2026-10-14

**Authors:** Ayman M. Atta, Hamad A. Al-Lohedan, Abdelrahman O. Ezzat

**Affiliations:** 1 Surfactants Research Chair, Department of Chemistry, College of Science, King Saud University, P.O. Box 2455, Riyadh 11451, Saudi Arabia; hlohedan@ksu.edu.sa (H.A.A.-L.); ao_ezzat@yahoo.com (A.O.E.); 2 Petroleum Application Department, Egyptian Petroleum Research Institute, Cairo 11727, Egypt

The journal retracts the article titled “Synthesis of Silver Nanoparticles by Green Method Stabilized to Synthetic Human Stomach Fluid” [1], cited above.

Following publication, concerns were brought to the attention of the Editorial Office regarding the integrity of images presented in this publication [1].

Adhering to our standard procedure, the Editorial Office and Editorial Board conducted an investigation that identified indications of inappropriate image editing and duplication of panels presented in Figures 3, 8, 9 and 11 published in [1], and duplications between Figure 8c presented in this article and Figure 4b published earlier in [2], produced by a similar authorship, presenting different experimental conditions. Although the authors cooperated during this investigation, the explanations and supporting materials provided were not deemed sufficient by the Editorial Board to resolve the identified concerns. Consequently, the Editorial Board has lost confidence in the reliability of the reported findings and has decided to retract this publication as per MDPI’s retraction policy.

This retraction was approved by the Editor-in-Chief of the journal *Molecules*.

The authors have been informed of this retraction but did not provide a comment on this decision.
